# Comparison of WhatsApp® and Face-to-Face case based learning in undergraduate dental students: A randomized controlled trial

**DOI:** 10.12669/pjms.40.11.9248

**Published:** 2024-12

**Authors:** Wasim Ijaz, Usman Mahboob, Humera Adeeb, Naveed Iqbal

**Affiliations:** 1 Wasim Ijaz, BDS, FCPS, MHPE Assistant Professor Orthodontics, Ayub College of Dentistry, Abbottabad, Pakistan; 2 Usman Mahboob, MBBS, MPH, FHEA, DHPE, Fellow FAIMER Professor Medical Education, Institute of Health Professions Education & Research, Khyber Medical University, Peshawar, Pakistan; 3 Humera Adeeb, MBBS, MPhil, MHPE Assistant Professor Medical Education, Institute of Health Professions Education & Research, Khyber Medical University, Peshawar, Pakistan; 4 Naveed Iqbal, BDS, MPH, MHPE Lecturer Dentistry, Ayub College of Dentistry, Abbottabad, Pakistan

**Keywords:** Case-based learning, Orthodontics, Undergraduate Dental Students, Teaching Tool, WhatsApp®

## Abstract

**Objective:**

The study compares two different interfaces of CBL (WhatsApp® and Face-to-Face) in undergraduate dental students and examines WhatsApp’s effectiveness as a teaching tool.

**Methods:**

A Randomized Controlled Trial (RCT) was conducted from May 2023 to October 2023 at Ayub College of Dentistry, Orthodontic Department, Abbottabad, involving 30 participants randomly assigned to control (Face-to-Face) Group-1 and experimental (WhatsApp®) Group-2. The six-week study utilized pre-validated questionnaires and Multiple-Choice Questions assessments, comparing post-test scores. Assessment of the intervention involved evaluating Kirkpatrick levels one and two, with responses being rated using a five-point Likert scale. Statistical analysis employed Students’ t-tests for group comparisons and paired t-tests for MCQ results before and after the intervention for each group. A p-value less than 0.05 indicated statistical significance, with SPSS version 23 used for analysis.

**Results:**

The study involved 30 participants with a mean age of 23.23±0.67 years, encompassing both males (53.3%) and females (46.7%). All survey participants (100%) used WhatsApp, highlighting its popularity. Everyone had internet access, with 73.3% using smartphones and 26.7% using laptops for mobile internet. Post-intervention, both control and experimental groups exhibited improved post-test scores (p<0.001), but the comparison between groups showed no statistically significant difference (p>0.50). Over 50% of students expressed satisfaction with WhatsApp as a teaching tool, resulting in a learning gain of 36.18%.

**Conclusions:**

The study concluded that both WhatsApp® and Face-to-Face CBL effectively improved dental students’ learning, with WhatsApp® yielding results comparable to traditional methods.

## INTRODUCTION

Numerous facets of human behavior and communication have undergone significant change as a result of information technology advancements. The effects on educational practices have been significant because of these changes. Mobile internet devices have become more widely available, especially in the last ten years, which has boosted the number of educational possibilities available outside of the traditional classroom.^1^ These portable internet-connected devices give medical learners access to social media networks^2^ and applications.^3^ Despite worries about a lack of evidence supporting this transformation, technological advancements have led to significant changes in educational methods, particularly with social media-based medical education activities.^4^ WhatsApp®, the widely used smartphone app, has become a convenient platform for medical education due to its ease of use, instant messaging, large chat groups, broadcast features, media exchange, and two-way information sharing.^5^

In medical universities globally, progressive interactive learning approaches have supplanted traditional lecture (TL) methods. Case-Based Learning (CBL), akin to problem-based learning (PBL), is a student-centered teaching approach adopted in this shift.^6^ CBL involves presenting students with real-world clinical cases, including comprehensive medical histories, physical assessments, diagnoses, and lab tests, encouraging them to approach these cases as challenges to enhance critical thinking abilities.^7^

CBL stands as a widely embraced orthodontic teaching methodology that incorporates real-world clinical scenarios to facilitate both theoretical and practical learning. Conventional (Face-to-Face) CBL, however, poses time constraints, hindering in-depth case analysis. Supplementing CBL with WhatsApp® addresses this issue, enabling more learning, active participation, discussions, higher test scores, and improved information retention, as demonstrated in previous studies.^6^ Additionally, WhatsApp® facilitates easy communication, allowing students to seek mentor assistance without hesitation.^8^

WhatsApp® serves as an educational tool, enabling information sharing and fostering discussion. It can function as a platform for CBL discussions (WhatsApp® CBL). While highlighting the benefits of instant messaging in medical education, additional research is required for validation. Additionally, no study has explored a comparison between two distinct interfaces of CBL (WhatsApp® and Face-to-Face) among undergraduate dental students. This study seeks to compare these two interfaces and assess WhatsApp’s effectiveness as a teaching tool in the context of undergraduate dental education.

## METHODS

**Fig.1:**
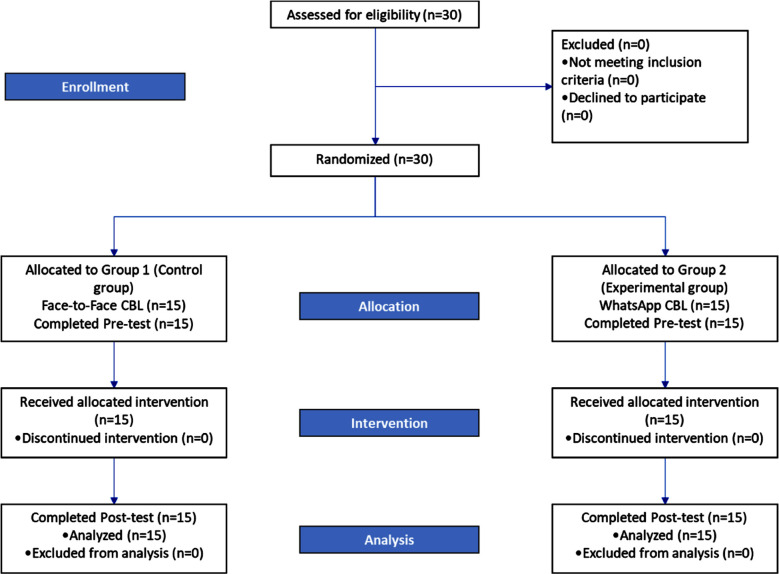
Consort Flow Diagram.

A Randomized Controlled Trial (Pretest-Posttest control group design) was conducted at the Ayub College of Dentistry, Orthodontic Department, Abbottabad, (AMC) from May 2023 to October 2023. The study included both male and female final-year BDS students. Exclusion criteria encompass students without WhatsApp® usage, those residing in remote areas without internet access, and students with below 60 percent marks. Using the probability sampling technique, a sample size of n=30 participants; n=15 participants in each group was calculated using an “Open-epi sample size calculator” with a 95% significance level and taking an expected percentage of exposed outcome i.e., 49% and Odds ratio 19 (Fig.1).^9^

### Ethical Approval:

It was obtained from the college ethical committee (Approval Code/ Ref. No. RC-EA-2023/100),

### RCT Registration:

This is a medical education intervention and as per ICMJE guidelines, does not come under clinical trials. It did not include any patients as participants nor was any drug tested in it. https://www.icmje.org/about-icmje/faqs/clinical-trials-registration/

After obtaining consent, 30 final years BDS students from Ayub Medical College Abbottabad were randomly divided into two groups (15 each) using a random number generator. To prevent gender differences between the groups, random allocation was carried out independently for male and female students. Over six weeks, Group-1 (control) underwent three Face-to-Face CBL sessions on malocclusion themes, whereas Group-2 (experimental) encountered similar sessions over WhatsApp® for the next three weeks. Both groups took a pretest before interventions. Each group received topics on malocclusion, using pre-designed clinical cases covering history, clinical examination, cephalometric analysis, and patient photos. Face-to-face sessions were 60 minutes, with the teacher facilitating case discussions. In WhatsApp® sessions, the same faculty monitored discussions after uploading the cases to their WhatsApp group, and students collaborated on answers before presenting via PowerPoint presentations in the classroom. A posttest with similar difficulty was conducted for both groups. The effectiveness of WhatsApp® as a teaching tool was assessed using the Barwood et al. procedure.^10^ Evaluation was done on Kirkpatrick levels one and two,^11^ and feedback on a five points Likert scale was obtained.^6^,^9^

Due to students’ first-hand involvement with either the intervention or control group, double-blinding was not feasible in this study. This study was single-blind by concealing the assigned groups’ identities from the faculty person administering the assessment. Content validation of the MCQ questionnaire, feedback questionnaire, and CBL sessions was done by sending e-mails to four orthodontic consultants. Face validity was done by eight BDS house officers. The MCQ questionnaire demonstrated a moderate level of internal consistency (Cronbach’s alpha= 0.75), while feedback questionnaires for WhatsApp® and Face-to-Face CBL showed moderate (0.75) and high (0.92) internal consistency, respectively. Descriptive statistics and t-tests were employed for data analysis in SPSS version 23, considering a p-value less than 0.05 as statistically significant.

## RESULTS

The mean age of the study’s 30 participants was 23.23±0.67 years. There were 14 (46.7%) female participants and 16 (53.3%) male participants in the study. All survey participants (100%) used WhatsApp, highlighting its popularity. Furthermore, they also acknowledged having access to the internet where they reside. Most participants (73.3%) utilized smartphones as their mobile internet devices, with laptops being used by the remaining 26.7%.

The paired samples test revealed significant increases in post-test scores for both control (5.20 to 7.86, t = -4.78, p < 0.001, Cohen’s d = 1.52) and experimental (4.93 to 8.53, t = -4.29, p < 0.001, Cohen’s d = 1.42) groups following intervention. A weak positive correlation (r = 0.219) in the experimental group suggested a link between higher pre-test scores and somewhat higher post-test scores. An independent samples test showed no significant difference in post-test scores between groups (p > 0.50, Cohen’s d = 0.25), indicating a marginal distinction, (Table-I).

**Table-I:** Group-wise Analysis of Pre-test and Post-test Means and Related Statistics.

Mean Comparison of Pre-test and Post-test on Control Group and Experimental Group									

	Pre-test		Post-test						

Variables	M	SD	M	SD	t	p*	r	Cohen’s d	
Control Group	5.20	1.37	7.86	2.06	-4.78	.001	.262	1.52	
Experimental Group	4.93	1.70	8.53	3.15	-4.29	.001	.219	1.42	

***Mean Comparison of Control and Experimental Groups on Post-test Score***									

	***Control Group***		***Experimental Group***						

***Variables***	***M***	***SD***	***M***	***SD***	***t***	p**			***Cohen’s d***

Post-test Score	7.86	2.06	8.53	3.15	-.68	.50			.25

*M* Mean *SD* Standard Deviation *t* t-statistics,

* Paired t-test

** Independent t-test *r* Correlation.

Overall learning gains as a result of the intervention were estimated using a procedure recommended by Barwood et al. Using the Barwood et al. procedure (see formula below), based on the pre-test and post-test scores, learning gains of 24.11% for the control and 36.18% for the experimental group were obtained.

### Formula:

Total Post-test Score obtained* - Total Pre-test Score obtained** × 100/ Maximum Score*** - Total Pre-test Score obtained = (118-84) × 100/ (225-84) = 24.11% (Control)

(128-73) × 100/ (225-73) = 36.18% (Experimental)

* Sum of individual post-test scores of all the participants

** Sum of individual pre-test scores of all the participants

*** Sum of all the items scores i.e., 15 × 15 = 225

Considering the responses, most participants (46.7% + 20.0% = 66.7%) were either satisfied or extremely satisfied using WhatsApp® as a teaching tool. Additionally, a sizable majority of participants (66.7% + 13.3% = 80.0%) either agreed or strongly agreed that using WhatsApp® as a teaching tool makes it easier for them to approach a teacher with questions. The survey results also indicated that most participants (46.7% + 20.0% = 66.7%) perceived time constraints as a problem during Face-to-Face CBL sessions, (Table-II).

**Table-II:** Feedback Evaluation Using a Likert Scale.

Statement	Strongly Disagree n (%)	Disagree n (%)	Neutral n (%)	Agree n (%)	Strongly Agree n (%)
Are you satisfied with this kind of teaching using WhatsApp as a teaching tool?	1 (6.7)	2 (13.3)	2 (13.3)	7 (46.7)	3 (20)
Do you think using WhatsApp as a teaching tool makes it easier for you to approach a teacher with a question?	0 (0.0)	1 (6.7)	2 (13.3)	10 (66.7)	2 (13.3)
Do you think that the time constraint was an issue for Face-to-Face CBL?	0 (0.0)	1 (6.7)	4 (26.7)	7 (46.7)	3 (20.0)

*n* Frequency % Percentage

## DISCUSSION

The increasing integration of digital tools into educational methodologies is reshaping how learning is delivered, particularly in fields like dental education where traditional face-to-face methods have long dominated. In this study, we explored the potential of WhatsApp®, a widely-used digital communication tool, as a platform for delivering case-based learning (CBL) sessions. Our findings highlight WhatsApp® as a promising tool that offers flexibility, accessibility, and adaptability, crucial factors for enhancing student engagement and learning outcomes in today’s digital age.

WhatsApp® utility as a teaching tool is supported by prior research that demonstrated the effectiveness of simple software for case-based e-learning.^12^ In our study, students expressed satisfaction with the convenience and ease of use provided by WhatsApp®, particularly appreciating its asynchronous nature, which allowed them to engage with the material and discussions at their own pace. This flexibility is especially valuable for final-year BDS students, who often juggle academic responsibilities with clinical practice.

Moreover, our findings align with an earlier study on hybrid learning, where WhatsApp® was integrated as an adjunct to traditional methods, resulting in positive impressions and improved academic performance.^13^ In our context, WhatsApp® was used to upload case scenarios, with discussions and conclusions drawn both online and during classroom sessions. This hybrid approach utilizes the strengths of both digital and face-to-face interactions, providing a balanced and comprehensive learning experience.

The growing use of smartphones as educational aids further emphasizes the relevance of incorporating tools such as WhatsApp® into the curriculum.^14^ As our study demonstrates, the strategic use of such digital platforms can enhance the learning experience by providing students with greater autonomy and opportunities for critical thinking and reflection, aspects that are sometimes constrained in traditional classroom settings.

In our study, students highlighted benefits such as flexibility and accessibility from online learning, echoing findings from previous studies.^6^,^15^ Furthermore, students expressed satisfaction with WhatsApp® ease and adaptability, enabling confident interaction with teachers. The asynchronous nature of WhatsApp® communication, noted in prior studies facilitated in-depth contemplation and critical thinking.^16^,^17^

Contrary to current research supporting the interactive advantages of Face-to-face CBL for instant feedback and real-time demonstrations, findings from an earlier study suggest that students prefer CBL discussions in small group tutorials over online conversations.^18^ Although appreciated for quick feedback, over 50% of our surveyed students identified time constraints as a drawback of Face-to-face CBL, highlighting a potential limitation in conventional classroom-based techniques.

We also found that both WhatsApp® and Face-to-Face CBL effectively improved student learning outcomes, and our results are consistent with other studies.^19^-^22^ These previous studies support our findings, reinforcing the effectiveness of CBL, whether delivered through WhatsApp® or Face-to-Face, in enhancing educational outcomes.

### Limitations:

This was a single-centre study that can be extended to other educational institutions to consider more variables such as educational resources, quality of teaching and difference in curriculum. Additionally, the long-term impacts of such intervention can be explored in future studies. The varying degrees of technology literacy among students could have influenced their perception and performance in the WhatsApp® learning strategy.

## CONCLUSION

We compared WhatsApp® and Face-to-Face CBL in undergraduate dental students and both teaching methods were demonstrated as effective in enhancing learning outcomes. WhatsApp® CBL emerges as a viable substitute with flexibility and satisfactory results. This underscores the potential benefits of integrating technological tools like WhatsApp® in dental education. However, the study emphasizes the need for a balanced approach between traditional and technology-based teaching. Despite its contributions, awareness of the study’s limitations is crucial. Further research is recommended to explore long-term consequences and optimize technology-enhanced learning in dental education.

### Disclosure:

This article presents findings from a Master of Health Professions Education (MHPE) thesis conducted by the primary author WI under the supervision of UM and co-supervision by HA.

## References

[1] Maudsley G, Taylor D, Allam O, Garner J, Calinici T, Linkman K (2019). A Best Evidence Medical Education (BEME) systematic review of:What works best for health professions students using mobile (hand-held) devices for educational support on clinical placements?BEME Guide No. 52. Med Teacher.

[2] Bullock A, Webb K (2015). Technology in postgraduate medical education:a dynamic influence on learning?. Postgrad Med J.

[3] Boruff JT, Storie D (2014). Mobile devices in medicine:a survey of how medical students, residents, and faculty use smartphones and other mobile devices to find information. J Med Lib Assoc.

[4] Coleman E, O'Connor E (2019). The role of WhatsApp®in medical education;a scoping review and instructional design model. BMC Med Educ.

[5] Mohanakrishnan K, Jayakumar N, Kasthuri A, Nasimuddin S, Malaiyan J, Sumathi G (2017). Whatsapp enhances medical education:is it the future?. Int J Med Sci Public Health.

[6] Grover S, Garg B, Sood N (2020). Introduction of case-based learning aided by WhatsApp messenger in pathology teaching for medical students. J Postgrad Med.

[7] Williams B (2005). Case based learning—a review of the literature:is there scope for this educational paradigm in prehospital education?. Emerg Med J.

[8] Martins JCS, de Lima JB, Cartaxo RO, Sette-de-Souza PH (2022). Use of WhatsApp in dental education:A scoping review. Med Sci Educ.

[9] Alhazmi A, Quadri MFA (2020). Comparing case-based and lecture-based learning strategies for orthodontic case diagnosis:A randomized controlled trial. J Dent Educ.

[10] Baseer N, Degnan J, Moffat M, Mahboob U (2020). Micro-feedback skills workshop impacts perceptions and practices of doctoral faculty. BMC Med Educ.

[11] Smidt A, Balandin S, Sigafoos J, Reed VA (2009). The Kirkpatrick model:A useful tool for evaluating training outcomes. J Intellect Dev Disabil.

[12] Woelber J, Hilbert T, RatkaKrüger P (2012). Can easytouse software deliver effective e-learning in dental education?A randomised controlled study. Eur J Dent.

[13] Alsharif AT, Alsharif B, Alsharif L, Althagafi N, Natto ZS, Kassim S (2020). Effectiveness of WhatsApp as a part of a hybrid learning environment:an opportunity for post-COVID-19 pandemic pedagogy. J Contemp Dent Pract.

[14] Junaid SM, Jamil B, Khan MA, Akbar Z, Shah S, Nadeem N (2023). Smartphone as an educational tool the perception of dental faculty members of all the dental colleges of Khyber Pakhtunkhwa-Pakistan. BMC Med Educ.

[15] Al-Amin M, Al Zubayer A, Deb B, Hasan M (2021). Status of tertiary level online class in Bangladesh:students'response on preparedness, participation and classroom activities. Heliyon.

[16] Menon RK, Seow LL (2021). Development of an Online Asynchronous Clinical Learning Resource (“Ask the Expert”) in Dental Education to Promote Personalized Learning. Healthcare.

[17] Hasan N, Khan NH (2020). Online teaching-learning during covid-19 pandemic:students'perspective. Turk Online J Dist Educ.

[18] Holland JC, Pawlikowska T (2019). Undergraduate medical students'usage and perceptions of anatomical case-based learning:Comparison of facilitated small group discussions and eLearning resources. Anat Sci Educ.

[19] Dong H, Guo C, Zhou L, Zhao J, Wu X, Zhang X (2022). Effectiveness of case-based learning in Chinese dental education:a systematic review and meta-analysis. BMJ Open.

[20] Bi M, Zhao Z, Yang J, Wang Y (2019). Comparison of case-based learning and traditional method in teaching postgraduate students of medical oncology. Med Teacher.

[21] Oliván-Blázquez B, Aguilar-Latorre A, Gascón-Santos S, Gómez-Poyato MJ, Valero-Errazu D, Magallón-Botaya R (2022). Comparing the use of flipped classroom in combination with problem-based learning or with case-based learning for improving academic performance and satisfaction. Act Learn High Educ.

[22] Yoo MS, Park HR (2015). Effects of case-based learning on communication skills, problem-solving ability, and learning motivation in nursing students. Nurs Health Sci.

